# SEOM-GEM clinical guidelines for cutaneous melanoma (2023)

**DOI:** 10.1007/s12094-024-03497-2

**Published:** 2024-05-15

**Authors:** Iván Márquez-Rodas, Eva Muñoz Couselo, Juan F. Rodríguez Moreno, Ana Mª Arance Fernández, Miguel Ángel Berciano Guerrero, Begoña Campos Balea, Luis de la Cruz Merino, Enrique Espinosa Arranz, Almudena García Castaño, Alfonso Berrocal Jaime

**Affiliations:** 1 Hospital General Universitario Gregorio Marañón, Madrid, Spain; 2 Hospital Vall d’Hebron & Vall d’Hebron Institute of Oncology (VHIO), Barcelona, Spain; 3 Instituto de Investigación Sanitaria HM Hospitales, Madrid, Spain; 4 Hospital Clínic, Barcelona, Spain; 5 Unidad Intercentros de Oncología. Hospitales Universitarios Regional y Virgen de la Victoria (HURyVV), Málaga, Spain; 6 Hospital Univ. Lucus Augusti, Lugo, Spain; 7 Cancer Immunotherapy, Biomedicine Institute of Seville (IBIS)/CSIC, Clinical Oncology Department, University Hospital Virgen Macarena and School of Medicine, University of Seville, Seville, Spain; 8 Hospital Univ. La Paz, CIBERONC, Madrid, Spain; 9 Hospital Univ. Marqués de Valdecilla, Santander, Spain; 10 Consorcio Hospital General Univ, Valencia, Spain

**Keywords:** Melanoma, Staging, Immunotherapy, Targeted therapy

## Abstract

Cutaneous melanoma incidence is rising. Early diagnosis and treatment administration are key for increasing the chances of survival. For patients with locoregional advanced melanoma that can be treated with complete resection, adjuvant—and more recently neoadjuvant—with targeted therapy—BRAF and MEK inhibitors—and immunotherapy—anti-PD-1-based therapies—offer opportunities to reduce the risk of relapse and distant metastases. For patients with advanced disease not amenable to radical treatment, these treatments offer an unprecedented increase in overall survival. A group of medical oncologists from the Spanish Society of Medical Oncology (SEOM) and Spanish Multidisciplinary Melanoma Group (GEM) has designed these guidelines, based on a thorough review of the best evidence available. The following guidelines try to cover all the aspects from the diagnosis—clinical, pathological, and molecular—staging, risk stratification, adjuvant therapy, advanced disease therapy, and survivor follow-up, including special situations, such as brain metastases, refractory disease, and treatment sequencing. We aim help clinicians in the decision-making process.

## Introduction

A constant evolution in melanoma management thrived since the advent of targeted therapies and modern immunotherapy, especially in adjuvant and advanced settings. The growing evidence needs to be updated to offer the best options available to the patients.

We wrote these guidelines after conducting a thorough review of the most relevant recently published translational and clinical studies. It provides the consensus of ten leading melanoma experts from the Spanish Multidisciplinary Melanoma Group (GEM), and the Spanish Society of Medical Oncology (SEOM), along with the external review panel of two experts designated by the SEOM. To assign levels of evidence and grades of recommendation, we used the Infectious Diseases Society of America-US Public Health Service Grading System for Ranking Recommendations in Clinical Guidelines. The systemic treatment recommendations take into consideration the reimbursement availability from the Spanish public health system.

## Incidence and epidemiology

Melanoma is a malignant tumor originating from melanocytes of the skin in up to 90% of cases. In 2020, there were 324,635 new cases of cutaneous melanoma, with wide geographic differences: age-standardized rates (ASR) range from 35,8 (60 cases per 100,000 people) in Australia and New Zealand to around 18 in the USA and Europe (25–30 cases per 100,000 people) or 0.3 in Africa and Asia [1].

In Spain, there is an estimation of 8049 new cases in 2023 [2]. The incidence has steadily increased in the past decades, as in most Western countries. This is mainly due to ultraviolet radiation (UVR) exposure from sunlight and/or indoor tanning, which represents the main risk factor. Other relevant risk factors are Fitzpatrick skin type I and II—pale, white skins with tan difficulties and sunburns, especially in childhood—high nevi count (> 100), atypical nevi, immunodeficiency, xeroderma pigmentosum, and personal or familial history of melanoma [1, 3].

As a worldwide public health concern, primary prevention measures are emphasized to reduce UVR exposure from sunbathing and indoor tanning, and to increase the use of sun protection and protective clothing [3] **(level of evidence 1, grade of recommendation A)**.

## Diagnosis, pathology, and molecular testing

Clinical analysis of suspicious lesions includes three aspects: the ABCD rule—Asymmetry, Border irregularities, Color heterogeneity, and Dynamics or evolution in the color, size, or elevation—the ugly duckling sign—the lesion is different from the rest in the same patient—and chronological analysis of changes [4]. Dermatoscopy by an experienced physician is recommended for the diagnosis of pigmented lesions [5] **(level of evidence 1, grade of recommendation A)**. All suspicious lesions must be confirmed histologically by excisional biopsy following the eighth edition of the American Joint Committee on Cancer (AJCC 8th edition) [6] (Table 1).

**Table 1:** Melanoma staging AJCC 8th edition [6]

T category	Thickness	Ulceration status
TX: Primary tumor thickness cannot be assessed (e.g., diagnosis by curettage)	Not applicable	Not applicable
T0: No evidence of primary tumor (e.g., unknown primary or completely regressed melanoma)	Not applicable	Not applicable
Tis (melanoma in situ)	Not applicable	Not applicable
T1	≤ 1.0 mm	Unknown or unspecified
T1a	< 0.8 mm	Without ulceration
T1b	< 0.8 mm	With ulceration
	0.8–1.0 mm	With or without ulceration
T2	> 1.0–2.0 mm	Unknown or unspecified
T2a	> 1.0–2.0 mm	Without ulceration
T2b	> 1.0–2.0 mm	With ulceration
T3	> 2.0–4.0 mm	Unknown or unspecified
T3a	> 2.0–4.0 mm	Without ulceration
T3b	> 2.0–4.0 mm	With ulceration
T4	> 4.0 mm	Unknown or unspecified
T4a	> 4.0 mm	Without ulceration
T4b	> 4.0 mm	With ulceration

N category	Number of tumor-involved regional lymph nodes	Presence of in-transit, satellite, and/or microsatellite metastases
NX	Regional nodes not assessed (e.g., sentinel lymph node biopsy not performed, regional nodes previously removed for another reason)	No
N0	No regional metastases detected	No
N1	One tumor-involved node or any number of in-transit, satellite, and/or microsatellite metastases with no tumor-involved nodes	
N1a	One clinically occult (i.e., detected by SLN biopsy)	No
N1b	One clinically detected	No
N1c	No regional lymph-node disease	Yes
N2	Two or three tumor-involved nodes or any number of in-transit, satellite, and/or microsatellite metastases with one tumor-involved node	
N2a	Two or three clinically occult (i.e., detected by SLN biopsy)	No
N2b	Two or three, at least one of which was clinically detected	No
N2c	One, clinically occult or clinically detected	Yes
N3	Four or more tumor-involved nodes or any number of in-transit, satellite, and/or microsatellite metastases with two or more tumor-involved nodes, or any number of matted nodes without or with in-transit, satellite, and/or microsatellite metastases	
N3a	Four or more clinically occult (i.e., detected by SLN biopsy)	No
N3b	Four or more, at least one of which was clinically detected, or the presence of any number of matted nodes	No
N3c	Two or more clinically occult or clinically detected and/or presence of any number of matted nodes	Yes

M category	Anatomic site	LDH level	
M0	No evidence of distant metastasis	Not applicable	
M1	Evidence of distant metastasis	See below	
M1a	Distant metastasis to skin, soft tissue including muscle, and/or non-regional lymph node	Not recorded or unspecified	
M1a (0)		Not elevated	
M1a (1)		Elevated	
M1b	Distant metastasis to lung with or without M1a sites of disease	Not recorded or unspecified	
M1b (0)		Not elevated	
M1b (1)		Elevated	
M1c	Distant metastasis to non-CNS visceral sites with or without M1a or M1b sites of disease	Not recorded or unspecified	
M1c (0)		Not elevated	
M1c (1)		Elevated	
M1d	Distant metastasis to CNS with or without M1a, M1b, or M1c sites of disease	Not recorded or unspecified	
M1d (0)		Not elevated	
M1d (1)		Elevated	
T	N	M	STAGE
Tis	N0	M0	0
T1A	N0	M0	IA
T1b–T2a	N0	M0	IB
T2b–T3a	N0	M0	IIA
T3b–T4a	N0	M0	IIB
T4b	N0	M0	IIC
Any T, Tis	> N1	M0	III
Any T	Any N	M1	IV

Several proteins are commonly used as markers for melanoma in immunohistochemistry testing: S-100 protein, SOX-10, HMB- 45, PRAME, and MART-1 [7].

Determination of *BRAF* V600 status is mandatory in patients with stage IV melanoma [8]. The same applies to earlier stages if treatment with targeted therapy is considered [9] **(level of evidence 1, grade of recommendation A)**. Determination of C-KIT [10] and *NRAS* status in stage IV disease is optional [11] **(level of evidence 2, grade of recommendation C)**.

Immunohistochemical determination of programmed death ligand 1 (PD-L1) is not mandatory because patients with negative expression may respond to anti-PD1 antibodies [12] **(level of evidence 1, grade of recommendation C**). Currently, in Spain, the expression of PD-L1 must be tested for grant access to combination with immunotherapy due to regulatory restrictions.

## Staging

A full body skin check by an experienced dermatologist and a complete physical examination is mandatory in all patients diagnosed with melanoma at any stage. In pT1b–pT4b melanomas, TNM staging with ultrasound (US) for locoregional lymph-node metastasis, and/or computed tomography (CT) or positron emission tomography (PET) scans and/or brain magnetic resonance imaging (MRI), could be recommended for proper tumour assessment. Serum lactate dehydrogenase (LDH) blood levels must also be obtained in all patients with metastatic melanoma **(level of evidence 3, grade of recommendation B)**. Table 1 summarizes AJJC 8th edition staging [6].

## Treatment of localized disease and regional lymph staging

### Treatment of primary tumors

Excisional biopsy, preferably with 1–3 mm negative margins, is indicated for any suspicious lesion **(level of evidence 5, grade of recommendation A)**. Upon pathological confirmation of the diagnosis, definitive surgery with wide margins is performed. The deep margin should extend to the fascia, whereas lateral margins will depend on Breslow thickness: 0.5 cm for in situ melanomas, 1 cm for tumors with a thickness of up to 2 mm, and 2 cm for a thickness > 2 mm **(level of evidence 2, grade of recommendation B)** [13].

### Sentinel lymph node biopsy

Sentinel lymph node biopsy is recommended for melanomas with Breslow thickness > 0.8 mm or < 0.8 mm with ulceration [14] **(level of evidence 2, grade of recommendation B)**.

### Complete lymph node dissection

For patients with positive sentinel lymph node biopsy, complete lymph node dissection could carry morbidity and shows no impact on survival [15] **(level of evidence 1, grade of recommendation D)**. However, the procedure could be recommended in case of clinically detected regional lymph nodes or for some selected cases after discussion in a multidisciplinary tumor board [16] **(level of evidence 4, grade of recommendation C)**. Resection of satellite or in-transit metastases could be considered in highly selected cases **(level of evidence 4, grade of recommendation D)**.

## Adjuvant therapy

### Adjuvant radiotherapy

It shows benefits for lymph-node field control in patients at high risk of lymph-node field relapse after therapeutic lymphadenectomy for metastatic melanoma, but not in overall survival or metastasis-free survival. Moreover, it increases the risk of regional toxicity, so it is no longer routinely recommended [17] **(level of evidence 1, grade of recommendation D)**. The role of radiotherapy for in-transit metastasis has not been established.

### Adjuvant targeted therapy

The phase 3 COMBI-AD trial involved patients with stage III *BRAF* V600 mutant melanoma—according to AJJC 7th edition and stage IIIA with a minimum lymph node involvement of 1 mm. Here, a one-year adjuvant dabrafenib plus trametinib treatment improved relapse-free survival (RFS) and distant metastasis-free survival (DMFS) compared with placebo, which was maintained over time [9]. Overall survival (OS) was prolonged with targeted therapy in the primary analysis, but a statistically significant benefit over placebo is yet to be confirmed [18]. Hence, dabrafenib and trametinib are recommended as one standard option for patients with completely resected stage III *BRAF*-mutated melanoma **(level of evidence 1, grade of recommendation A).** *This treatment indication is not financed by the Spanish public health system, at the time of writing this document.*

To date, there is no trial showing the benefit of targeted therapy in the adjuvant setting for stage II or stage IV melanoma.

### Adjuvant immunotherapy

In patients with resected stage IIIB–IIIC–IV melanoma, nivolumab improved RFS and DMFS compared to ipilimumab. However, there were no differences in OS, with a toxicity profile in favor of nivolumab [19]. Pembrolizumab also improved RFS and DMFS versus placebo in patients with resected stage III [20]. The value of adjuvant immunotherapy in patients at lower risk of relapse—stage IIIA—is controversial, as subgroup analysis did not reveal significant differences. Both nivolumab and pembrolizumab can be considered as options in high risk for relapse resected melanoma—IIIA–IIIC pembrolizumab, IIIB–IV nivolumab—regardless of *BRAF* status **(level of evidence 1, grade of recommendation A)**. *These treatments for resected stage III are only reimbursed by the Spanish public health system for specific stages IIIC–D*.

Combination immunotherapy cannot be recommended in the adjuvant setting. A trial comparing nivolumab plus low-dose ipilimumab versus nivolumab alone did not show an improvement in disease-free survival for patients with resected stages IIIB–IIIC–IIID–IV [21] **(level of evidence 1, grade of recommendation E)**.

The optimal selection of adjuvant therapy for patients with stage III *BRAFV600* mutant melanoma remains unclear. Comorbidities, the risk and types of toxicities, and the patient’s preferences should be considered **(level of evidence 5, grade of recommendation C)**.

The KEYNOTE-716 study found that a one-year treatment with pembrolizumab improved both RFS and DMFS versus placebo in patients with stage IIB and IIC resected melanoma [22, 23]. The CHECKMATE 76 K trial also included patients in stage IIB/IIC, randomized 2:1 to placebo or a one-year treatment with nivolumab. Here, nivolumab increased RFS and DMFS after the treatment [24]. Both nivolumab and pembrolizumab can be considered as treatment options for stage IIB–C resected melanoma **(level of evidence 1, grade of recommendation A)**. Only *pembrolizumab is currently financed for stage IIB–C melanoma by the Spanish public health system at the time of writing this manuscript*. Table 2 summarizes the main results of randomized pivotal clinical trials in the adjuvant setting.

**Table 2:** Pivotal clinical trials in the adjuvant setting

Clinical trial	Population and key features	ARMS (N)	RFS (95% CI)	DMFS (95% CI)	OS (95% CI)
COMBI-AD [9]	AJCC 7 Stage III, complete resected *BRAF*-mutated melanoma. IIIA must be > 1 mm ganglionic tumor burden and with complete lymphadenectomy	Dabrafenib and trametinib (DT) (438) vs. placebo (432), 1 year	Median (months) NR DT (47.9–NR) vs. 16.6 (12.7–22.1) placebo	HR 0.55 (0.44–0.7)	Not reported
			HR 0.51 (0.42–0.61)		
			At year 5, 52% (48–58) DT vs. 36% (32–41) placebo	At year 5, 65% (61–71) in DT vs. 54% (49–60) placebo	
KEYNOTE-054 [20]	AJCC 7 Stage III, complete resected melanoma (regardless *BRAF*). IIIA must be > 1 mm ganglionic tumor burden and complete lymphadenectomy	Pembrolizumab (pem) (514) vs. placebo (505), 1 year	HR 0.59 (0.49–0.70)	HR 0.60 (0.49–0.73)	Not reported
			At year 3.5, 59.8% (55.3–64.1) pem vs. 41.4% (37–45.8) placebo	At year 3.5, 65.3% (60.9–69.5) pem vs. 49.4% (44.8–53.8) placebo	
CHECKMATE 238 [19]	AJCC 7 Stage IIIB–IV, complete resected melanoma (regardless *BRAF*)	Nivolumab (nivo) (453) vs. ipilimumab (ipi) (453), 1 year	Median (months) 61 (42.5‒NR) nivo versus 24.1 (16.6‒35.1) ipi	Median NR in either group	Median NR in either group
			HR 0.72 (0.60‒0.86)	HR, 0.79 (0.63‒0.99)	HR 0.86 (0.66‒1.12)
			At year 5, 50% nivo vs. 39% ipi	At year 5, 58% nivo vs. 51% ipi	At year 5, 76% nivo vs. 72% ipi
KEYNOTE-716 [22, 23]	AJCC 7 Stage IIB–C, complete resected melanoma (regardless *BRAF*)	Pembrolizumab (pem) (487) vs. placebo (489), 1 year	Median (months) 37.2 (NR–NR) pem vs. NR (NR-NR) placebo	Median NR in either group	Not reported
			HR 0.64 (0.50–0.84)	HR 0.64 (0.47–0.88)	
			At 24 months, 81% (77–85) pem vs. 73% (68–77) placebo	At 24 months, 88% (84–91) pem vs. 82% (78–86) placebo	
CHECKMATE 76 K [24]	AJCC 7 Stage IIB–C, complete resected melanoma (regardless *BRAF*)	Nivolumab (nivo) (526) vs. placebo (264), 1 year	Median (months) NR (28.5–NR) nivo vs. NR (21.6–NR) placebo	Not reported	Not reported
			HR 0.42 (0.30–0.59)		
			At 12 months, 89% (86–92) nivo vs. 79% (74–84) placebo		

## Neoadjuvant therapy

Especially with immunotherapy, it is gaining attention in the melanoma community due to its potential to improve results of adjuvant therapy. In the phase 2 SWOG1801 trial, patients with resectable stages IIIB–IV were randomized to receive three cycles of pembrolizumab followed by surgery and completion of pembrolizumab up to one year, versus surgery followed by one year of pembrolizumab. Although the trial did not demonstrate an impact in OS, event-free survival at 2 years was 72% (CI 64–80) in the neoadjuvant-adjuvant group versus 49% (CI 41–59) in the control-adjuvant group. Thus, it makes neoadjuvant therapy a feasible option for these patients [25] **(level of evidence 2, grade of recommendation B)**. There is ongoing clinical research to determine the optimal immunotherapy neoadjuvant strategy.

## Treatment of oligometastatic disease

One-third of patients with resected metastasis may become long-term survivors. So, for patients with resectable oligometastatic disease, surgical excision or stereotactic radiosurgery (SRS) should be considered whenever feasible, preferentially combined with adjuvant systemic therapies [26] **(level of evidence 3, grade of recommendation B)**.

## Treatment of advanced metastatic disease: targeted therapy for *BRAF-*mutated melanoma

The CO-BRIM trial studied the combination of vemurafenib and cobimetinib. The study demonstrated increased progression-free survival (PFS) and OS over vemurafenib monotherapy [27].

The COMBI-v and COMBI-D trials compared the combination of dabrafenib and trametinib to vemurafenib and dabrafenib monotherapy. Both demonstrated improved efficacy of combination therapy over BRAF inhibition alone [28–30].

The COLUMBUS trial has demonstrated a similar benefit in PFS and OS with the combination of encorafenib and binimetinib over vemurafenib [31].

Any of these three combinations of BRAF and MEK inhibitors can be the therapy of choice when targeted therapy is considered **(level of evidence 1, grade of recommendation A).** When selecting the combination, patient preferences, drug availability, and efficiency criteria should be considered **(level of evidence 5, grade of recommendation C).**

## Treatment of advanced metastatic disease: immunotherapy

CTLA-4 blocker ipilimumab was the first treatment to show an improvement in OS of patients with metastatic melanoma [32], and in combination with chemotherapy over chemotherapy alone [33]. However, PD-1 inhibitors—such as nivolumab [12], pembrolizumab [34], or the combination of ipilimumab plus nivolumab [12]— are preferred due to better outcomes in response rate (ORR), PFS and OS, regardless of *BRAF* or PDL-1 status. Nivolumab plus relatlimab has demonstrated an improvement in PFS over nivolumab [35], but not in OS, after a median follow-up over 19 months [36].

Hence, for immunotherapy, anti-PD-1 monotherapy or combined with anti-CTLA-4 or anti-LAG-3 are the preferred options **(level of evidence 1, grade of recommendation A)** regardless of *BRAF* or PD-L1 status. The low-dose ipilimumab regimen—1 mg/kg ipilimumab plus 3 mg/kg nivolumab—presents less grade 3–5 toxicity than the pivotal CHECKMATE 067 regimen—ipilimumab 3 mg/kg plus nivolumab 1 mg/kg. For this reason, it is an option to consider when toxicity is a concern [37] **(level of evidence 1, grade of recommendation C)**.

*At the time of writing this document, ipilimumab and nivolumab reimbursement by the Spanish public health system is restricted to patients with PD-L1-negative melanoma, metastatic to the brain, or uveal melanoma. Nivolumab and relatlimab is not financed by the Spanish public health system at the time of writing this document.* Table 3 summarizes the main characteristics of the pivotal trials of targeted therapy and immunotherapy in advanced melanoma.

**Table 3:** Pivotal targeted therapy and anti-PD-1-based immunotherapy clinical trials

Clinical trial	Population and key features	Arms (N)	PFS (95% CI)	OS (95% CI)
Immunotherapy				
CHECKMATE 067 [12]	First-line advanced melanoma. 31.5% *BRAF* mutant Not designed for direct comparison between nivolumab and nivolumab plus ipilimumab	Ipilimumab and nivolumab (IPINIVO) (314) or nivolumab (NIVO) (316) vs. ipilimumab (IPI) (315)	Median (months) 11.5 (8.7–19.3) IPINIVO and 6.9 (5.1–10.2) NIVO vs. 2.9 (2.8–3.2) IPI	Median (months) > 60 (38.2–NR) IPINIVO and 36.9 (28.2–58.7) NIVO vs. 19.9 (16.8–24.6) IPI
			At year 5, 36% IPINIVO and 29% NIVO vs. 8% IPI	At year 5, 52% IPINIVO and 44% NIVO vs. 26% IPI
KEYNOTE-006 [34]	First- and second-line advanced melanoma. 34% second-line treatment 36% *BRAF* mutant	Pembrolizumab (in two different doses) (PEM) (556) vs. ipilimumab (IPI) (278)	Median (months) 8.4 (6.6–11.3) PEM vs. 3.4 (2.9–4.2) IPI	Median (months) 32.7 (24.5–41.6) PEM vs. 15.9 (13.3–22) IPI
			At year 4, 23% for PEM vs. 7.3% IPI	At year 5, 38.7% PEM vs. 31% IPI
RELATIVITY-047 [35, 36]	First-line advanced melanoma. 38.3% *BRAF* mutant	Nivolumab and relatlimab (NIVORELA) (355) vs. nivolumab (NIVO) (359)	Median (months) 10.1 (6.4–15.7) NIVORELA vs. 4.6 (3.4–5.6) NIVO	Median (months) NR (34.2–NR) NIVORELA vs. 34.1 (25.2–NR) NIVO
Targeted therapy				
CO-BRIM [27]	First-line *BRAF*-mutated advanced melanoma	Vemurafenib and cobimetinib (VC) (247) vs vemurafenib and placebo (V) (248)	Median (months) 12.6 (9.5–14.8) VC vs. 7.2 (5.6–7.5) V	Median (months) 22.5 (20.3–28.8) VC vs. 17.4 (15–19.8) V
			At year 5, 14% (9–19) VC vs. 10% (6–14) V	At year 5, 31% (25–37) VC vs. 26% (20–32) V
COMBI-v and Combi D [28]	Pooled analysis of two phase 3 clinical trials, first-line *BRAF*-mutated advanced melanoma	Dabrafenib and trametinib (DT) 563 vs. vemurafenib (V) (352) or dabrafenib and placebo (D) (212)	Median (months) 11.1 (9.5–12.8) DT vs. 8.8 (8–13.9) D vs. 7.3 V	Median (months) 25.9 (22.6–31.5) DT vs. 18.7 (15.2–23.7) D vs. 17.2 V
			At year 5, 19% (15–22) DT vs. 13% D vs. 9% V	At year 5, 34% (30–38) DT vs. 27% D vs. 23% V
COLUMBUS [31]	First-line (or second-line after immunotherapy) *BRAF*-mutated advanced melanoma	Encorafenib and binimetinib (EB) (192) vs. vemurafenib (191) vs encorafenib (E) (194) (part 1)	Median (months) 14.9 (11–20.2) EB vs 7.3 (5.6–7.9) for V vs. 9.6 (7.4–14.8) E	Median (months) 33.6 (24.4–39.2) EB vs 16.9 (14–24.5) V vs. 23.5 (19.6–33.6) E
			At year 5, 23% EB vs. 10% V vs. 19% E	At year 5, 35% EB vs. 21% V vs. 35% E

## Selection of first-line therapy in *BRAF*-mutant melanoma

DREAMseq is a phase 3 clinical trial that included 256 patients with advanced *BRAF*-mutant melanoma. They were randomized 1:1 with two drug sequences: dabrafenib and trametinib followed, after progression, by ipilimumab and nivolumab, versus the opposite sequence. This trial demonstrated that patients who started with ipilimumab and nivolumab had better two-year PFS (41.9% vs. 19.2%) and two-year OS (71.8% vs. 51.5%) [38].

Similarly, the phase 2 randomized clinical trial SECOMBIT compared these two sequences using instead encorafenib and binimetinib as targeted therapy. A “sandwich” third arm consisted of encorafenib and binimetinib for two months, followed by ipilimumab and nivolumab. The two-year OS rates were 65% in arm A—encorafenib plus binimetinib followed by ipilimumab plus nivolumab upon progression, 73% in arm B —ipilimumab plus nivolumab until progression followed by encorafenib plus binimetinib, and 69% in arm C—sandwich approach [39].

When choosing immunotherapy versus targeted therapy for BRAF-mutant melanoma in the advanced setting, ipilimumab and nivolumab could be a better option over targeted therapy. However, there is no current prospective evidence of anti-PD-1 in monotherapy strategy over targeted therapy **(level of evidence 2, grade of recommendation B)**.

Thus, the selection of first-line therapy for patients with metastatic disease is often based on the patient profile—comorbidities, ECOG, symptoms, and life expectancy—and the melanoma features—tumor burden, site of metastasis, and LDH level [40]. It is also important to consider the patient's preference for the oral or intravenous treatment option, and the expected toxicity profile of each therapeutic option **(level of evidence 4, grade of recommendation C)**.

Finally, triple combinations of BRAF, MEK, and PD-1 inhibitors, have not demonstrated a significant impact in terms of OS over targeted therapy, but a higher level of toxicity **(level of evidence 1, grade of recommendation D)** [41, 42]. *These combinations are not approved by the European Medicines Agency (EMA) for melanoma and are not reimbursed by the Spanish public health system, at the time of writing this document.*

**Fig. 1:**
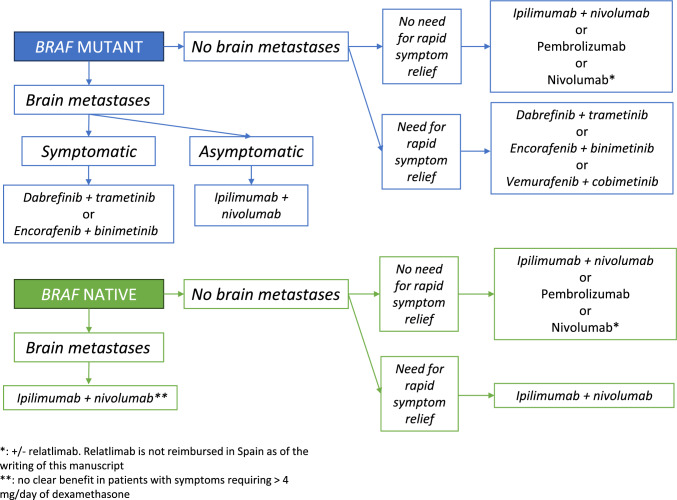
Proposed first line algorithm for metastatic melanoma

Figure 1 proposes an algorithm for the first-line treatment decision, according to BRAF mutation status, patient, and disease features.

## Treatment of advanced metastatic disease: second line and beyond

Election of subsequent therapies is based in diverse variables like performance status, comorbidities, or results of prior treatments. Inclusion in clinical trial should be strongly considered in this setting **(level of evidence 5, grade of recommendation A)**.

### Immunotherapy

For patients previously treated with PD-1 inhibitors, combinations of checkpoint inhibitors showed limited efficacy but remain an option for some patients. In the randomized phase 2 SWOG1616 clinical trial, ipilimumab plus nivolumab improve ORR and PFS versus ipilimumab monotherapy (28% vs. 9% for ORR; median 3 vs. 2.7 months for PFS). While there is no significant impact in OS [43], both ipilimumab with or without nivolumab are valid options in the anti-PD-1 refractory setting **(level of evidence 2, grade of recommendation B)**.

Cellular therapy using tumor-infiltrating lymphocytes (TILs) shows a benefit in PFS and a trend in OS compared with ipilimumab in patients with anti-PD-1 refractory melanoma (median 7.2 months for TILs vs. 3.1 for ipilimumab) [44]. Safety issues, such as the need of initial metastasectomy for TIL generation, and the use of lymphodepleting chemotherapy regimens or high-dose Interleukin-2 (IL-2) after TIL infusion, require that patients are carefully selected **(level of evidence 2, grade of recommendation C)**.

In patients with accessible lesions, low tumor burden and without a rapidly progressive disease, intralesional drugs alone or in combination with systemic immunotherapy could be considered. Oncolytic virus, talimogene laherparepvec (T-VEC), was mainly active in stage IIIB–IVM1a patients with mild toxicity and durable responses **(level of evidence 5, grade of recommendation C)** [45].

High-dose IL-2 has a significant toxicity with modest results in efficacy, but patients who achieve a complete response (less than 10%) tend to have durable responses and high rates of long-term survival. Its use should be restricted to institutions with experience and in selected cases [46] **(level of evidence 3, grade of recommendation D).*** None of these treatments (TILs, TVEC of HD-IL-2) are reimbursed by the Spanish public health system at the time of writing this document.*

### Chemotherapy and other targeted therapies

Chemotherapy is a feasible option beyond immunotherapy and/or targeted therapy in metastatic melanoma when no further options exist. However, currently, there are no randomized clinical trials available. Most of the evidence reported is based on retrospective studies or analysis of subsequent lines in clinical trials for first- or second-line treatment [47] **(level of evidence 4, grade of recommendation C)**.

Molecular screening helps identify patients who could potentially benefit from targeted therapy, mainly in the clinical trial setting. For example, the presence of *KIT* mutations is more common in acral melanoma. Imatinib and nilotinib were tested in patients with metastatic melanomas with a *KIT* mutation or amplification, demonstrating an acceptable ORR and disease control rate [48, 49]. Unfortunately, most of these responses were limited in duration **(level of evidence 3, grade of recommendation C)**. *This treatment is not approved by the* European Medicines Agency* (EMA) and is not reimbursed by the Spanish public health system at the time of writing this document.*

### Treatment beyond progression and rechallenge

Treatment beyond progression—i.e., immunotherapy when a pseudoprogression is suspected—and rechallenge—i.e., re-exposure of immunotherapy or targeted therapy after a variable treatment-free interval—might be options in selected patients. Both may be usable based on retrospective data in targeted therapy and immunotherapy [50, 51] **(level of evidence 4, grade of recommendation C)**.

## Local and systemic treatment for patients with brain metastases

Melanoma brain metastases (MBM) frequently exist at diagnosis or develop during the disease. Therapeutic value of neurosurgical resection of a single BM with no evidence of systemic disease remains well established [52] **(level of evidence 2, grade of recommendation A)**. Stereotactic radiosurgery (SRS) on the surgical cavity is recommended after excision of BMs [53] **(level of evidence 1, grade of recommendation A)**. Whole brain radiotherapy (WBRT) is discouraged in brain metastases not amenable to SRS, and in leptomeningeal disease **(level of evidence 5, grade of recommendation D)**.

The COMBI-MB study evaluated the combination of dabrafenib with trametinib in patients with *BRAF*-mutant MBM [54]. The study reported a response rate of 58% in asymptomatic untreated BMs, quite alike to the response rate in patients with symptomatic BM. Median PFS was 5.6 months, almost half (5.6 vs. 10.1 months) compared to that observed with the same treatment in patients with extracranial disease. This suggests an earlier treatment failure in the brain. Similar results have been found with encorafenib and binimetinib [55] **(level of evidence 3, grade of recommendation C)**.

Triple therapy with vemurafenib, cobimetinib, and atezolizumab in BRAFV600 mutant MBM demonstrated an intracranial response rate was 42% [56] **(level of evidence 3, grade of recommendation C)**. *This treatment combination is not approved by the European Medicines Agency (EMA) and is not reimbursed by the Spanish public health system at the time of writing this report.*

SRS can be used as a salvage strategy in cases of local progression of patients treated with BRAF and MEK inhibitors, despite overall disease control **(level of evidence 5, grade of recommendation B)**. It is advisable to stop targeted therapy during WBRT, while this does not seem necessary during SRS **(level of evidence 5, grade of recommendation A)**.

The activity of ipilimumab in combination with nivolumab was evaluated in two phase 2 studies. One showed intracranial responses (51 to 54%) in patients with asymptomatic MBM [57]. In the other, median PFS was not reached after a follow-up of 34.3 months [58]. However, this combination demonstrated limited efficacy in patients with symptomatic metastases or receiving steroid therapy [58]. Data suggest that ipilimumab plus nivolumab is the preferred first-line option for patients with asymptomatic MBM, irrespective of *BRAF* status **(level of evidence 3, grade of recommendation A)** if there is no contraindication for immunotherapy.

We need prospective randomized clinical trial results to better delineate the optimal association of immunotherapy and radiotherapy for patients with MBM.

## Follow-up

Despite most melanomas being diagnosed in early stages, 20–30% of these patients may develop a recurrence within 5 years [59]. For this, a multidisciplinary follow-up of melanoma patients after surgical treatment of the primary lesion is recommended. From primary care, and through all the specialties that treat each patient, clinical recommendations must be unified. Patient education can increase compliance with sun protection and allow skin and lymph node self-examinations to detect recurrence [60] **(level of evidence 5, grade of recommendation B)**.

The frequency of clinical examination is not well established. A sensible approach might be the higher the staging, the more frequent the follow-up [60] **(level of evidence 5, grade of recommendation B)**.

Biomarkers have been examined for clinical utility in melanoma, but few have been validated or approved for clinical use. They include LDH, S-100, and circulating tumor DNA [61]. Therefore, routine blood tests are optional [62] **(level of evidence 4, grade of recommendation C)**.

The role of imaging in the follow-up of high-risk melanoma patients is increasingly relevant, given the availability of effective immunotherapies and targeted therapies. Early detection of tumour recurrence could be associated with an OS benefit. A real-world investigation with stage IIB–IIIC patients undergoing imaging surveillance compared treatment and survival outcomes between asymptomatic surveillance-detected recurrence (ASDR) and symptomatic recurrence. ASDR relapse (45% of cases) was associated with a lower burden of disease at recurrence, better prognosis, higher response rates to systemic treatment, and improved survival outcomes [63] **(level of evidence 4, grade of recommendation B)**. However, the optimal radiological techniques of choice—i.e., CT scan, PET–CT scan, brain MRI—remain unknown.

Lymph node sonography must be performed regularly in patients with stage III melanomas—i.e., every tree to six months for the first 2 years, and every six months for the next 3 years. This is especially relevant in patients with positive sentinel lymph nodes without lymph node dissection ^15^**(level of evidence 1, grade of recommendation A)**.

For earlier stages I–IIA, where the risk of relapse is lower, radiological follow-up with CT scan and brain MRI is optional **(level of evidence 5, grade of recommendation C)**.

*Additional table. Summary of clinical recommendations and their level of evidence*.

**Table:** 

Statement	Level of evidence	Grade of recommendation
Primary prevention—the use of sun protection and protective clothes—is emphasized to reduce UVR exposure	1	A
Dermatoscopy by an experienced physician is recommended for the diagnosis of pigmented lesions	1	A
Determination of *BRAF* V600 status is mandatory in patients with stage IV melanoma and III if targeted therapy is considered for adjuvant setting	1	A
Determination of *C-KIT* and *NRAS* status in stage IV disease is optional	2	C
Determination of PD-L1 is not mandatory because cases with negative expression can respond to anti-PD1 antibodies	1	C
In pT1b–pT4b melanoma, ultrasound (US) for locoregional lymph-node metastasis, and/or computed tomography (CT) or positron emission tomography (PET) scans and brain magnetic resonance imaging (MRI), could be recommended for proper tumour assessment	3	B
Excisional biopsy preferably with a 1–3 mm negative margins is indicated for any suspicious lesion	5	A
Lateral margins will depend on Breslow thickness: 0.5 cm for in situ melanomas, 1 cm for tumors with thickness of up to 2 mm, and 2 cm for > 2 mm	2	B
Sentinel lymph node biopsy is recommended for melanomas with Breslow > 0.8 mm of thickness or < 0.8 mm with ulceration, i.e., melanomas with stage ≥ IB of the AJCC 8th edition classification	2	B
Systematic complete lymph-node dissection is not recommended in all patients with positive SLN	1	D
Complete lymph-node dissection could be recommended in the case of clinically detected regional lymph-node metastases or after discussion in multidisciplinary tumour board	4	C
Resection of satellite or in-transit metastases could be considered in highly selected cases	4	D
Adjuvant radiotherapy is no longer routinely recommended	1	D
Dabrafenib and trametinib is recommended for patients with completed resected stage III *BRAF*-mutated melanoma	1	A
Both nivolumab and pembrolizumab are recommended in high risk for relapse resected melanoma (III–IV) regardless *BRAF* status	1	A
Both nivolumab and pembrolizumab can be considered as options in resected melanoma IIB–C stages	1	A
Neoadjuvant pembrolizumab for three cycles before surgery is a feasible option for patients with resectable stages IIIB–IV melanoma	2	B
Dabrafenib plus trametinib, vemurafenib plus cobimetinib or encorafenib plus binimetinib should be the therapy of choice when targeted therapy is considered	1	A
When selecting the targeted therapy combination, patient preferences, drug availability, and efficiency criteria should be considered	5	C
When immunotherapy is considered, anti PD-1 in monotherapy or combined with anti CTLA-4 or anti LAG-3 is the preferred option	1	A
When choosing immunotherapy versus targeted therapy for *BRAF*-mutant melanoma in the advanced setting, ipilimumab and nivolumab could be a better option over targeted therapy	2	B
The low-dose ipilimumab with standard nivolumab dose regimen could be an option to consider when toxicity is a concern	1	C
Selection of first-line therapy for patients with *BRAF*-mutant metastatic disease is often based on the patient profile and patient's preferences	4	C
Triple combination of BRAF, MEK and PD(L)-1 cannot be recommended due to low benefit/risk balance	1	D
Inclusion in clinical trials should be strongly considered in the anti-PD-1 refractory setting	5	A
Ipilimumab with or without nivolumab are options for patients with anti-PD-1 refractory disease	2	B
TILs are options for patients with anti-PD-1 refractory disease, with careful patient selection	2	C
Oncolytic virus T-VEC can be an option in very selected patients with oligometastatic disease	5	C
High-dose IL-2 should be restricted to institutions with experience and very selected cases	3	D
Chemotherapy is a feasible option beyond immunotherapy and/or targeted therapy in metastatic melanoma when no further options exist	4	C
Imatinib or nilotinib could be an option in metastatic melanomas with *KIT* mutation	3	C
Treatment beyond progression and rechallenge might be options in selected patients, both in targeted therapy and in immunotherapy	4	C
Surgery of solitary brain metastases is an accepted option, especially when the systemic disease is controlled	2	A
Stereotactic radiosurgery (SRS) on the surgical cavity is recommended after excision of BMs	1	A
Whole brain radiotherapy (WBRT) is discouraged in brain metastases not amenable to SRS and in leptomeningeal disease	5	D
Dabrafenib and trametinib or encorafenib and binimetinib are acceptable options for patients with *BRAF*-mutant melanoma metastatic to the brain, especially when immunotherapy has failed or is contraindicated	3	C
Triplet therapy with vemurafenib plus cobimetinib plus atezolizumab is an acceptable option for patients with *BRAF*-mutant melanoma metastatic to the brain	3	C
SRS can be used as a rescue strategy in patients treated with BRAF and MEK inhibitors in cases of local progression	5	B
It is advisable to stop targeted therapy during WBRT, while this seems not to be necessary with SRS	5	A
Ipilimumab plus nivolumab is the preferred first-line option for patients with asymptomatic BM, irrespective of *BRAF* status	3	A
Multidisciplinary follow-up of melanoma patients after surgical treatment of the primary lesion is recommended	5	B
The frequency of clinical examination is not well established, being the higher the staging, the more frequent the follow-up a sensible approach	5	B
Blood biomarkers have not been validated or approved for clinical use	4	C
Early detection of tumour recurrence could be associated with an OS benefit. However, the optimal radiological technique of choice remains unknown	4	B
Lymph-node sonography in patients with stage III melanomas must be performed regularly especially in patients with positive sentinel lymph nodes without lymph node dissection	1	A
For earlier stages I-IIA where the risk of relapse is lower, radiological follow-up with CT scan and brain MRI is optional	5	C

## References

[1] Huang J, Chan SC, Ko S, Lok V, Lin Z, Xu L, et al. Global incidence, mortality, risk factors and trends of melanoma: a systematic analysis of registries. Am J Clin Dermatol. 2023;24(6):965–75. 10.1007/s40257-023-00795-3. 37296344 10.1007/s40257-023-00795-3

[2] El cáncer en cifras | SEOM: Sociedad Española de Oncología Médica. https://seom.org/prensa/el-cancer-en-cifras. Accessed 16 Sep 2023.

[3] Long GV, Swetter SM, Menzies AM, Gershenwald JE, Scolyer RA. Cutaneous melanoma. Lancet Lond Engl. 2023;402(10400):485–502. 10.1016/S0140-6736(23)00821-8. 10.1016/S0140-6736(23)00821-8 37499671

[4] Garbe C, Amaral T, Peris K, Arenberger P, Bastholt L, Bataille V, et al. European consensus-based interdisciplinary guideline for melanoma. Part 2: treatment—Update 2019. Eur J Cancer Oxf Engl. 1990;2020(126):159–77. 10.1016/j.ejca.2019.11.015. 10.1016/j.ejca.2019.11.015 31866016

[5] Kittler H, Pehamberger H, Wolff K, Binder M. Diagnostic accuracy of dermoscopy. Lancet Oncol. 2002;3(3):159–65. 10.1016/s1470-2045(02)00679-4. 11902502 10.1016/s1470-2045(02)00679-4

[6] Gershenwald JE, Scolyer RA, Hess KR, Sondak VK, Long GV, Ross MI, et al. Melanoma staging: evidence-based changes in the American Joint Committee on Cancer eighth edition cancer staging manual. Cancer J Clin. 2017;67(6):472–92. 10.3322/caac.21409. 10.3322/caac.21409 PMC5978683 29028110

[7] Saleem A, Narala S, Raghavan SS. Immunohistochemistry in melanocytic lesions: updates with a practical review for pathologists. Semin Diagn Pathol. 2022;39(4):239–47. 10.1053/j.semdp.2021.12.003. 35016807 10.1053/j.semdp.2021.12.003

[8] Chapman PB, Hauschild A, Robert C, Haanen JB, Ascierto P, Larkin J, et al. Improved survival with vemurafenib in melanoma with BRAF V600E mutation. N Engl J Med. 2011;364(26):2507–16. 10.1056/NEJMoa1103782. 21639808 10.1056/NEJMoa1103782 PMC3549296

[9] Dummer R, Hauschild A, Santinami M, Atkinson V, Mandalâ M, Kirkwood JM, et al. Five-year analysis of adjuvant dabrafenib plus trametinib in stage III melanoma. N Engl J Med. 2020;383(12):1139–48. 10.1056/NEJMoa2005493. 32877599 10.1056/NEJMoa2005493

[10] Meng D, Carvajal RD. KIT as an oncogenic driver in melanoma: an update on clinical development. Am J Clin Dermatol. 2019;20(3):315–23. 10.1007/s40257-018-0414-1. 30707374 10.1007/s40257-018-0414-1

[11] Dummer R, Schadendorf D, Ascierto PA, Arance A, Dutriaux C, Di Giacomo AM, et al. Binimetinib versus dacarbazine in patients with advanced NRAS-mutant melanoma (NEMO): a multicentre, open-label, randomised, phase 3 trial. Lancet Oncol. 2017;18(4):435–45. 10.1016/S1470-2045(17)30180-8. 28284557 10.1016/S1470-2045(17)30180-8

[12] Larkin J, Chiarion-Sileni V, Gonzalez R, Grob J-J, Rutkowski P, Lao CD, et al. Five-Year Survival with Combined Nivolumab and Ipilimumab in Advanced Melanoma. N Engl J Med. 2019;381(16):1535–46. 10.1056/NEJMoa1910836. 31562797 10.1056/NEJMoa1910836

[13] Hayes AJ, Maynard L, Coombes G, Newton-Bishop J, Timmons M, Cook M, et al. Wide versus narrow excision margins for high-risk, primary cutaneous melanomas: long-term follow-up of survival in a randomised trial. Lancet Oncol. 2016;17(2):184–92. 10.1016/S1470-2045(15)00482-9. 26790922 10.1016/S1470-2045(15)00482-9 PMC4737890

[14] El Sharouni MA, Stodell MD, Ahmed T, Suijkerbuijk KPM, Cust AE, Witkamp AJ, et al. Sentinel node biopsy in patients with melanoma improves the accuracy of staging when added to clinicopathological features of the primary tumor. Ann Oncol Off J Eur Soc Med Oncol. 2021;32(3):375–83. 10.1016/j.annonc.2020.11.015. 10.1016/j.annonc.2020.11.015 33253862

[15] Faries MB, Thompson JF, Cochran AJ, Andtbacka RH, Mozzillo N, Zager JS, et al. Completion dissection or observation for sentinel-node metastasis in melanoma. N Engl J Med. 2017;376(23):2211–22. 10.1056/NEJMoa1613210. 28591523 10.1056/NEJMoa1613210 PMC5548388

[16] Morton DL, Wanek L, Nizze JA, Elashoff RM, Wong JH. Improved long-term survival after lymphadenectomy of melanoma metastatic to regional nodes. Analysis of prognostic factors in 1134 patients from the John Wayne Cancer Clinic. Ann Surg. 1991;214(4):491–9. 10.1097/00000658-199110000-00013. (**discussion 499-501**). 1953101 10.1097/00000658-199110000-00013 PMC1358554

[17] Burmeister BH, Henderson MA, Ainslie J, Aimslie J, Fisher R, di Julio J, et al. Adjuvant radiotherapy versus observation alone for patients at risk of lymph-node field relapse after therapeutic lymphadenectomy for melanoma: a randomised trial. Lancet Oncol. 2012;13(6):589–97. 10.1016/S1470-2045(12)70138-9. 22575589 10.1016/S1470-2045(12)70138-9

[18] Long GV, Hauschild A, Santinami M, Atkinson V, Mandalà M, Chiarion-Sileni V, et al. Adjuvant dabrafenib plus trametinib in stage III BRAF-mutated melanoma. N Engl J Med. 2017;377(19):1813–23. 10.1056/NEJMoa1708539. 28891408 10.1056/NEJMoa1708539

[19] Larkin J, Del Vecchio M, Mandalá M, Gogas H, Arance Fernandez AM, Dalle S, et al. Adjuvant nivolumab versus ipilimumab in resected stage III/IV melanoma: 5-year efficacy and biomarker results from CheckMate 238. Clin Cancer Res Off J Am Assoc Cancer Res. 2023;29(17):3352–61. 10.1158/1078-0432.CCR-22-3145. 10.1158/1078-0432.CCR-22-3145 PMC10472092 37058595

[20] Eggermont AMM, Blank CU, Mandalà M, Long GV, Atkinson VG, Dalle S, et al. Adjuvant pembrolizumab versus placebo in resected stage III melanoma (EORTC 1325-MG/KEYNOTE-054): distant metastasis-free survival results from a double-blind, randomised, controlled, phase 3 trial. Lancet Oncol. 2021;22(5):643–54. 10.1016/S1470-2045(21)00065-6. 33857412 10.1016/S1470-2045(21)00065-6

[21] Weber JS, Schadendorf D, Del Vecchio M, Larkin J, Atkinson V, Schenker M, et al. Adjuvant therapy of nivolumab combined with ipilimumab versus nivolumab alone in patients with resected stage IIIB-D or stage IV melanoma (CheckMate 915). J Clin Oncol Off J Am Soc Clin Oncol. 2023;41(3):517–27. 10.1200/JCO.22.00533. 10.1200/JCO.22.00533 PMC9870220 36162037

[22] Luke JJ, Rutkowski P, Queirolo P, Del Vecchio M, Mackiewicz J, Chiarion-Sileni V, et al. Pembrolizumab versus placebo as adjuvant therapy in completely resected stage IIB or IIC melanoma (KEYNOTE-716): a randomised, double-blind, phase 3 trial. Lancet Lond Engl. 2022;399(10336):1718–29. 10.1016/S0140-6736(22)00562-1. 10.1016/S0140-6736(22)00562-1 35367007

[23] Long GV, Luke JJ, Khattak MA, de la Cruz ML, Del Vecchio M, Rutkowski P, et al. Pembrolizumab versus placebo as adjuvant therapy in resected stage IIB or IIC melanoma (KEYNOTE-716): distant metastasis-free survival results of a multicentre, double-blind, randomised, phase 3 trial. Lancet Oncol. 2022;23(11):1378–88. 10.1016/S1470-2045(22)00559-9. 36265502 10.1016/S1470-2045(22)00559-9

[24] Kirkwood JM, Del Vecchio M, Weber J, Hoeller C, Grob J-J, Mohr P, et al. Adjuvant nivolumab in resected stage IIB/C melanoma: primary results from the randomized, phase 3 CheckMate 76K trial. Nat Med. 2023;29(11):2835–43. 10.1038/s41591-023-02583-2. 37845511 10.1038/s41591-023-02583-2 PMC10667090

[25] Patel SP, Othus M, Chen Y, Wright GP Jr, Yost KJ, Hyngstrom JR, et al. Neoadjuvant-Adjuvant or Adjuvant-Only Pembrolizumab in Advanced Melanoma. N Engl J Med. 2023;388(9):813–23. 10.1056/NEJMoa2211437. 36856617 10.1056/NEJMoa2211437 PMC10410527

[26] Ch’ng S, Uyulmaz S, Carlino MS, Pennington TE, Shannon TE, Rtshiladze M, et al. Re-defining the role of surgery in the management of patients with oligometastatic stage IV melanoma in the era of effective systemic therapies. Eur J Cancer Oxf Engl 1990. 2021;153:8–15. 10.1016/j.ejca.2021.04.037. 10.1016/j.ejca.2021.04.037 34126335

[27] Ascierto PA, Dréno B, Larkin J, Ribas A, Liszkay G, Maio M, et al. 5-Year outcomes with cobimetinib plus vemurafenib in BRAFV600 Mutation-positive advanced melanoma: extended follow-up of the coBRIM Study. Clin Cancer Res Off J Am Assoc Cancer Res. 2021;27(19):5225–35. 10.1158/1078-0432.CCR-21-0809. 10.1158/1078-0432.CCR-21-0809 PMC9401485 34158360

[28] Robert C, Grob JJ, Stroyakovskiy D, Karaszewska B, Hauschild A, Levchenko E, et al. Five-year outcomes with dabrafenib plus trametinib in metastatic melanoma. N Engl J Med. 2019;381(7):626–36. 10.1056/NEJMoa1904059. 31166680 10.1056/NEJMoa1904059

[29] Robert C, Karaszewska B, Schachter J, Rutkowski P, Mackiewicz A, Stroiakovski D, et al. Improved overall survival in melanoma with combined dabrafenib and trametinib. N Engl J Med. 2015;372(1):30–9. 10.1056/NEJMoa1412690. 25399551 10.1056/NEJMoa1412690

[30] Long GV, Stroyakovskiy D, Gogas H, Levchenko E, de Graud F, Larkin J, al,. Dabrafenib and trametinib versus dabrafenib and placebo for Val600 BRAF-mutant melanoma: a multicentre, double-blind, phase 3 randomised controlled trial. Lancet Lond Engl. 2015;386(9992):444–51. 10.1016/S0140-6736(15)60898-4. 10.1016/S0140-6736(15)60898-4 26037941

[31] Dummer R, Flaherty KT, Robert C, Arance A, de Groot JWB, Garbe C, et al. COLUMBUS 5-year update: a randomized, open-label, phase III trial of encorafenib plus binimetinib versus vemurafenib or encorafenib in patients With BRAF V600-mutant melanoma. J Clin Oncol Off J Am Soc Clin Oncol. 2022;40(36):4178–88. 10.1200/JCO.21.02659. 10.1200/JCO.21.02659 PMC9916040 35862871

[32] Hodi FS, O’Day SJ, McDermott DF, Weber RW, Sosman JA, Haanen JB, et al. Improved survival with ipilimumab in patients with metastatic melanoma. N Engl J Med. 2010;363(8):711–23. 10.1056/NEJMoa1003466. 20525992 10.1056/NEJMoa1003466 PMC3549297

[33] Maio M, Grob JJ, Aamdal S, Bondarenko I, Robert C, Thomas L, et al. Five-year survival rates for treatment-naive patients with advanced melanoma who received ipilimumab plus dacarbazine in a phase III trial. J Clin Oncol Off J Am Soc Clin Oncol. 2015;33(10):1191–6. 10.1200/JCO.2014.56.6018. 10.1200/JCO.2014.56.6018 PMC5795709 25713437

[34] Robert C, Ribas A, Schachter J, Arance A, Grob J-J, Mortier L, et al. Pembrolizumab versus ipilimumab in advanced melanoma (KEYNOTE-006): post-hoc 5-year results from an open-label, multicentre, randomised, controlled, phase 3 study. Lancet Oncol. 2019;20(9):1239–51. 10.1016/S1470-2045(19)30388-2. 31345627 10.1016/S1470-2045(19)30388-2

[35] Tawbi HA, Schadendorf D, Lipson EJ, Ascierto PA, Matamala L, Castillo Gutiérrez E, et al. Relatlimab and nivolumab versus nivolumab in untreated advanced melanoma. N Engl J Med. 2022;386(1):24–34. 10.1056/NEJMoa2109970. 34986285 10.1056/NEJMoa2109970 PMC9844513

[36] Long GV, Stephen Hodi F, Lipson EJ, Schadenforf D, Ascierto PA, Matamala L, et al. Overall survival and response with nivolumab and relatlimab in advanced melanoma. NEJM Evid. 2023;2(4):EVIDo2200239. 10.1056/EVIDoa2200239. 10.1056/EVIDoa2200239 38320023

[37] Lebbé C, Meyer N, Mortier L, Marquez-Rodas I, Robert C, Rutkowski P, et al. Evaluation of two dosing regimens for nivolumab in combination with ipilimumab in patients with advanced melanoma: results from the phase IIIb/IV CheckMate 511 Trial. J Clin Oncol. 2019;37(11):867–75. 10.1200/JCO.18.01998. 30811280 10.1200/JCO.18.01998 PMC6455714

[38] Atkins MB, Lee SJ, Chmielowski B, Tarhini AA, Cohen GI, Truong T-G, et al. Combination dabrafenib and trametinib versus combination nivolumab and ipilimumab for patients with advanced BRAF-mutant melanoma: the DREAMseq Trial-ECOG-ACRIN EA6134. J Clin Oncol Off J Am Soc Clin Oncol. 2023;41(2):186–97. 10.1200/JCO.22.01763. 10.1200/JCO.22.01763 PMC9839305 36166727

[39] Ascierto PA, Mandalà M, Ferrucci PF, Guidoboni M, Rutkowski P, Ferraresi V, et al. Sequencing of ipilimumab plus nivolumab and encorafenib plus binimetinib for untreated BRAF-mutated metastatic melanoma (SECOMBIT): a randomized, three-arm, open-label phase ii trial. J Clin Oncol Off J Am Soc Clin Oncol. 2023;41(2):212–21. 10.1200/JCO.21.02961. 10.1200/JCO.21.02961 36049147

[40] Long GV, Grob JJ, Nathan P, Ribas A, Robert C, Schadendorf D, et al. Factors predictive of response, disease progression, and overall survival after dabrafenib and trametinib combination treatment: a pooled analysis of individual patient data from randomised trials. Lancet Oncol. 2016;17(12):1743–54. 10.1016/S1470-2045(16)30578-2. 27864013 10.1016/S1470-2045(16)30578-2

[41] Ascierto PA, Stroyakovskiy D, Gogas H, Robert C, Lewis K, Protsenko S, et al. Overall survival with first-line atezolizumab in combination with vemurafenib and cobimetinib in BRAFV600 mutation-positive advanced melanoma (IMspire150): second interim analysis of a multicentre, randomised, phase 3 study. Lancet Oncol. 2023;24(1):33–44. 10.1016/S1470-2045(22)00687-8. 36460017 10.1016/S1470-2045(22)00687-8

[42] Dummer R, Long GV, Robert C, Tawbi HA, Flaherty KT, Ascierto PA, et al. Randomized Phase III Trial evaluating spartalizumab plus dabrafenib and trametinib for BRAF V600-mutant unresectable or metastatic melanoma. J Clin Oncol Off J Am Soc Clin Oncol. 2022;40(13):1428–38. 10.1200/JCO.21.01601. 10.1200/JCO.21.01601 PMC9061149 35030011

[43] VanderWalde A, Bellasea SL, Kendra KL, Khushalani NI, Campbell KM, Scumpia PO, et al. Ipilimumab with or without nivolumab in PD-1 or PD-L1 blockade refractory metastatic melanoma: a randomized phase 2 trial. Nat Med. 2023;29(9):2278–85. 10.1038/s41591-023-02498-y. 37592104 10.1038/s41591-023-02498-y PMC10708907

[44] Rohaan MW, Borch TH, van den Berg JH, Met O, Kessels R, Geukes Foppen MH, et al. Tumor-Infiltrating Lymphocyte Therapy or Ipilimumab in Advanced Melanoma. N Engl J Med. 2022;387(23):2113–25. 10.1056/NEJMoa2210233. 36477031 10.1056/NEJMoa2210233

[45] Andtbacka RHI, Collichio F, Harrington KJ, Middleton MR, Downey G, Ӧhrling K, et al. Final analyses of OPTiM: a randomized phase III trial of talimogene laherparepvec versus granulocyte-macrophage colony-stimulating factor in unresectable stage III-IV melanoma. J Immunother Cancer. 2019;7(1):145. 10.1186/s40425-019-0623-z. 31171039 10.1186/s40425-019-0623-z PMC6554874

[46] Atkins MB, Kunkel L, Sznol M, Rosenberg SA. High-dose recombinant interleukin-2 therapy in patients with metastatic melanoma: long-term survival update. Cancer J Sci Am. 2000;6(Suppl 1):S11-14. 10685652

[47] Gupta A, Gomes F, Lorigan P. The role for chemotherapy in the modern management of melanoma. Melanoma Manag. 2017;4(2):125–36. 10.2217/mmt-2017-0003. 30190915 10.2217/mmt-2017-0003 PMC6094602

[48] Guo J, Carvajal RD, Dummer R, Hauschild A, Daud A, Bastian BC, et al. Efficacy and safety of nilotinib in patients with KIT-mutated metastatic or inoperable melanoma: final results from the global, single-arm, phase II TEAM trial. Ann Oncol Off J Eur Soc Med Oncol. 2017;28(6):1380–7. 10.1093/annonc/mdx079. 10.1093/annonc/mdx079 PMC5452069 28327988

[49] Guo J, Si L, Kong Y, Xu X, Zhu Y, Corless CL, et al. Phase II, open-label, single-arm trial of imatinib mesylate in patients with metastatic melanoma harboring c-Kit mutation or amplification. J Clin Oncol Off J Am Soc Clin Oncol. 2011;29(21):2904–9. 10.1200/JCO.2010.33.9275. 10.1200/JCO.2010.33.9275 21690468

[50] Valpione S, Carlino MS, Mangana J, Mooradian MJ, McArthur G, Schadendorf D, et al. Rechallenge with BRAF-directed treatment in metastatic melanoma: a multi-institutional retrospective study. Eur J Cancer Oxf Engl. 1990;2018(91):116–24. 10.1016/j.ejca.2017.12.007. 10.1016/j.ejca.2017.12.007 29360604

[51] Beaver JA, Hazarika M, Mulkey F, Mushti S, Chen H, Sridhara R, et al. Patients with melanoma treated with an anti-PD-1 antibody beyond RECIST progression: a US Food and Drug Administration pooled analysis. Lancet Oncol. 2018;19(2):229–39. 10.1016/S1470-2045(17)30846-X. 29361469 10.1016/S1470-2045(17)30846-X PMC5806609

[52] Patchell RA, Tibbs PA, Walsh JW, Dempsey RJ, Maruyana Y, Kryscio RJ, et al. A randomized trial of surgery in the treatment of single metastases to the brain. N Engl J Med. 1990;322(8):494–500. 10.1056/NEJM199002223220802. 2405271 10.1056/NEJM199002223220802

[53] Brown PD, Ballman KV, Cerhan JH, Anderson SK, Carrero XW, Whitton AC, et al. Postoperative stereotactic radiosurgery compared with whole brain radiotherapy for resected metastatic brain disease (NCCTG N107C/CEC·3): a multicentre, randomised, controlled, phase 3 trial. Lancet Oncol. 2017;18(8):1049–60. 10.1016/S1470-2045(17)30441-2. 28687377 10.1016/S1470-2045(17)30441-2 PMC5568757

[54] Davies MA, Saiag P, Robert C, Grob J-J, Flaherty KT, Arance A, et al. Dabrafenib plus trametinib in patients with BRAFV600-mutant melanoma brain metastases (COMBI-MB): a multicentre, multicohort, open-label, phase 2 trial. Lancet Oncol. 2017;18(7):863–73. 10.1016/S1470-2045(17)30429-1. 28592387 10.1016/S1470-2045(17)30429-1 PMC5991615

[55] Marquez-Rodas I, Arance A, Guerrero MAB, Díaz Beveridge R, Alamo MDC, Garcia Castaño A, et al. 1038MO Intracranial activity of encorafenib and binimetinib followed by radiotherapy in patients with BRAF mutated melanoma and brain metastasis: preliminary results of the GEM1802/EBRAIN-MEL phase II clinical trial. Ann Oncol. 2021;32:S870. 10.1016/j.annonc.2021.08.1423.

[56] Dummer R, Queirolo P, Gerard Duhard P, Hu Y, Wang D, Jobim de Azevedo S, et al. Atezolizumab, vemurafenib, and cobimetinib in patients with melanoma with CNS metastases (TRICOTEL): a multicentre, open-label, single-arm, phase 2 study. Lancet Oncol. 2023. 10.1016/S1470-2045(23)00334-0. 37459873 10.1016/S1470-2045(23)00334-0

[57] Long GV, Atkinson V, Lo S, Sandhu S, Guminski AD, Brown MP, et al. Combination nivolumab and ipilimumab or nivolumab alone in melanoma brain metastases: a multicentre randomised phase 2 study. Lancet Oncol. 2018;19(5):672–81. 10.1016/S1470-2045(18)30139-6. 29602646 10.1016/S1470-2045(18)30139-6

[58] Tawbi HA, Forsyth PA, Hodi FS, Algazi AP, Hamid O, Lao CD, et al. Long-term outcomes of patients with active melanoma brain metastases treated with combination nivolumab plus ipilimumab (CheckMate 204): final results of an open-label, multicentre, phase 2 study. Lancet Oncol. 2021;22(12):1692–704. 10.1016/S1470-2045(21)00545-3. 34774225 10.1016/S1470-2045(21)00545-3 PMC9328029

[59] Rockberg J, Amelio JM, Taylor A, Jörgensen L, Ragnhammar P, Hansson J. Epidemiology of cutaneous melanoma in Sweden-Stage-specific survival and rate of recurrence. Int J Cancer. 2016;139(12):2722–9. 10.1002/ijc.30407. 27563839 10.1002/ijc.30407

[60] Campos-Balea B, Fernández-Calvo O, García-Figueiras R, Neira C, Peña-Penabad C, Rodríguez-López C, et al. Follow-up of primary melanoma patients with high risk of recurrence: recommendations based on evidence and consensus. Clin Transl Oncol Off Publ Fed Span Oncol Soc Natl Cancer Inst Mex. 2022;24(8):1515–23. 10.1007/s12094-022-02822-x. 10.1007/s12094-022-02822-x 35349041

[61] Lee RJ, Gremel G, Marshall A, Myers KA, Fisher N, Dunn JA, et al. Circulating tumor DNA predicts survival in patients with resected high-risk stage II/III melanoma. Ann Oncol Off J Eur Soc Med Oncol. 2018;29(2):490–6. 10.1093/annonc/mdx717. 10.1093/annonc/mdx717 PMC5834029 29112704

[62] Garbe C, Paul A, Kohler-Späth H, Ellwanger U, Stroebel W, Schwarz M, et al. Prospective evaluation of a follow-up schedule in cutaneous melanoma patients: recommendations for an effective follow-up strategy. J Clin Oncol Off J Am Soc Clin Oncol. 2003;21(3):520–9. 10.1200/JCO.2003.01.091. 10.1200/JCO.2003.01.091 12560444

[63] Ibrahim AM, Le May M, Bossé D, Marginean H, Song X, Nessim C, et al. Imaging intensity and survival outcomes in high-risk resected melanoma treated by systemic therapy at recurrence. Ann Surg Oncol. 2020;27(10):3683–91. 10.1245/s10434-020-08407-8. 32363515 10.1245/s10434-020-08407-8

